# Supplementary material for: Clinical manifestations and outcome of patients with primary amoebic meningoencephalitis in Pakistan. A single-center experience
Source: PLoS One. 2023 Nov 8;18(11):e0290394. doi: 10.1371/journal.pone.0290394 (PMC10631667; doi:10.1371/journal.pone.0290394)
Supplement: Attachment — Submitted filename: Reviewer Comments to Author_02.05.2023.docx [file pone.0290394.s001.docx]

# Reviewer Comments to Author

First of all, I want to thank to all of the authors of the manuscript. This study needs to be revised with more detailed and proper written language.

*Naegleria fowleri*, the causative agent of primary amoebic meningoencephalitis (PAM), is a free-living amoeba. It is a water-borne infection usually detected in children and young people with healthy immune systems who swim, dive and perform activities in fresh and hot springs. It is an important study in terms of addressing the Clinical Symptoms and Outcomes of Patients with Primary Amoebic Meningoencephalitis in Pakistan. The points mentioned below need to be considered in more detail.

- "*Naegleria fowleri*" or "*Naegleria*" needs to be corrected in italics throughout the manuscript.
- -In line 56, it is stated that the transmission route of *N. fowleri* can not only be through contaminated water, such as swimming in freshwater lakes, rivers, canals, and swimming pools, but also "dry infection" with dust in the air. It would be better to mention this transmission route here.
- In line 93, it should also mention the decrease in consciousness about the clinical symptoms that are the backbone of the study.
- The term "GCS" is not mentioned anywhere in the manuscript.
- In line 102, the spelling of "2 gm/day" should be corrected.
- In line 104, explaining the drug treatment complex administered for a surviving patient in more detail would be more beneficial.
- It is interesting t that 27 patients were positive by microscopy, while only ten were positive by PCR. Besides, the PCR method is more sensitive and specific, and PCR results are essential in confirming the microscopy. It would be better if you could explain how you evaluate this situation.
- Cerebral edema is 66.7% in line 114 and 66.6% in the table; whichever is correct should be corrected.
- Looking at the table, tonsil herniation (23.1%) is among the most common ones.
- "While imaging results showed higher tendency of cerebral edema among female patients with frequency of 60.6% vs. 100.0%; p=0.081 for female and male patients, respectively" in line 122 This sentence should be reviewed. Because while 100% cerebral edema is seen in women in table 3, it is expressed as 60.6% in men.
- In the discussion section, the clinical symptoms of the patients and the treatment options given should be discussed in more detail. For example, it should be discussed compared to clinical symptoms and treatments in a single case (https://doi.org/10.1007/s11686-021-00514-0).
